# Heritability of *Plasmodium* Parasite Density in a Rural Ugandan Community

**DOI:** 10.4269/ajtmh.2010.10-0049

**Published:** 2010-11-05

**Authors:** Rachel L. Pullan, Hasifa Bukirwa, Robert W. Snow, Simon Brooker

**Affiliations:** Department of Infectious and Tropical Diseases, London School of Hygiene and Tropical Medicine, London, United Kingdom; Uganda Malaria Surveillance Project, Mulago Hospital, Kampala, Uganda; Malaria Public Health and Epidemiology Group, Kenya Medical Research Institute/Wellcome Trust Research Programme, Nairobi, Kenya; Centre for Tropical Medicine, Nuffield Department of Clinical Medicine, University of Oxford, Oxford, United Kingdom

## Abstract

Many factors influence variation in *Plasmodium* infection levels, including parasite/host genetics, immunity, and exposure. Here, we examine the roles of host genetics and exposure in determining parasite density, and test whether effects differ with age. Data for 1,711 residents of an eastern Ugandan community were used in pedigree-based variance component analysis. Heritability of parasite density was 13% (*P* < 0.001) but was not significant after controlling for shared household. Allowing variance components to vary between children (< 16 years) and adults (≥ 16 years) revealed striking age differences; 26% of variation could be explained by additively acting genes in children (*P* < 0.001), but there was no genetic involvement in adults. Domestic environment did not explain variation in children and explained 5% in adults (*P* = 0.09). Genetic effects are an important determinant of parasite density in children in this population, consistent with previous quantitative genetic studies of *Plasmodium* parasitaemia, although differences in environmental exposure play a lesser role.

## Introduction

Genetic variation among individuals is known to profoundly affect susceptibility to malaria, with a growing number of genes identified as conferring resistance against infection.^1^–^3^ However, there are many complexities inherent in studying genetic susceptibility and resistance to malaria, not least is the range of phenotypes involved, and the overall contribution of host genetics relative to environmental factors remains poorly understood. For example, although a number of studies have suggested familial involvement in susceptibility to severe malaria^4^,^5^ and antibody responses to specific malaria antigens,^6^ the study designs used made the effects of genetics and shared domestic exposure inseparable. More recently, a study of children on the Kenyan coast reported that genetic effects and unidentified household effects each explained around one-quarter of phenotypic variance for both incidence of mild clinical malaria and hospital admission with malaria.^7^ Likewise, longitudinal studies of rural Sri Lankan and Thai populations have reported heritability of between 12% and 24% for levels of blood-stage parasitaemia during clinical malaria.^8^,^9^

In terms of both vaccine and drug development and evolutionary biology, severe clinical malaria has been the phenotype of interest,^2^ and as such, fewer genetic epidemiology studies have focused on asymptomatic blood-parasite densities. Studies have, however, suggested links between several red blood cell polymorphisms and reduced parasite rates/densities,^10^–^15^ although others have been contradictory.^16^–^21^ Meanwhile, segregation analyses from a series of longitudinal family studies of parasitaemia in Cameroon and Burkina Faso have provided clear evidence of complex genetic involvement^22^–^24^ and have suggested linkage to the major histocompatibility complex (MHC) and 5q31-33 regions.^25^–^27^ However, in terms of total genetic contribution, the only studies to report heritability for asymptomatic parasite densities have been unable to account for shared household effects. They did, however, show significant heritability of between 10% and 33% in Tanzanian and Senegalese populations.^28^,^29^

We previously showed significant household clustering of malaria parasitaemia among a rural community living in an area of high (stable) transmission in eastern Uganda.^30^ Here, we extend this analysis and use standard quantitative genetic methods to evaluate the roles played by shared domestic environment and additive host genetics in determining density of *Plasmodium* parasitaemia in this community. To separate these effects, we incorporate information on all known genetic relationships within and between houses, allowing robust estimation of the heritability of blood-stage infection levels. Bivariate variance-component models are then used to determine whether familial, household, and environmental involvements vary according to the age of the host.

## Materials and Methods

### Study area.

A cross-sectional study was conducted between June and December 2008 in four contiguous villages in Mulanda subcounty, Tororo District in eastern Uganda, a region hyperendemic for malaria.^31^ Government malaria-control initiatives include the promotion of intermittent preventive treatment during pregnancy and the distribution of insecticide-treated nets through antenatal care services. No additional campaigns have been implemented in the 2 years before this study. Residents are primarily Japadhola, a subgroup of the Luo who originally settled in the Budama region of eastern Uganda in the early 17th century; a small number also come from other Ugandan groups, including Iteso.

### Recruitment and procedures.

Further details of the study area and procedures are presented elsewhere.^30^ In brief, a census and socio-economic questionnaire were used for all households in the subcounty between June and August 2008. Household locations were mapped using an eTrex global positioning system (Garmin Ltd., Olathe, KS). Four representative villages were subsequently selected for the total population cross-sectional parasitological survey. Investigators met with elected government representatives and community leaders to inform them of the study and explain the methodology, and enumerated households were revisited. All resident adults and parents of children had the purpose of the study explained to them in the language with which they felt most comfortable, and signed informed consent was obtained for all participants. The study protocol was approved by the Makerere University Faculty of Medicine Research and Ethics Committee (#2008-043), Uganda National Council of Science and Technology (#HS 476), and London School of Hygiene and Tropical Medicine Ethics Committee (#5261).

A standardized questionnaire was administered to all adults and primary caregivers of children to record details of medical history and protective behaviors. A single finger-prick blood sample was obtained from all enrolled individuals for preparation of thick films for quantification of malaria parasite density using light microscopy. Parasite densities were calculated by blinded counting of the number of asexual parasites per 200 leukocytes (or per 500 leukocytes, if the count is < 10 asexual parasites/200 leukocytes), assuming a leukocyte count of 8,000/μL by two independent microscopists. Parasite density was reported as the arithmetic mean of the two readings. In addition, a rapid diagnostic test for malaria (OptiMAL; DiaMed, Cressier, Switzerland) was performed on all participants with fever (temperature > 37.2°C) or reported history of fever in the previous 24 hours. Those with a positive test but no evidence of danger signs of severe malaria were diagnosed with uncomplicated malaria and treated with artemether-lumefanthrine (20 mg artemether/120 mg lumefantrine, Coartem; Novartis Pharma, Basel, Switzerland) in accordance with national guidelines. Participants found to be suffering from danger signs of severe malaria were referred to the local health facility for case management. Thin films from a subsample of 20% of parasite-positive participants were read for species identification, showing that 94.0% were *Plasmodium falciparum* and 6.0% were *P. malariae*; no other species or mixed infections were seen.

Pedigrees were constructed by interviewing the primary caregiver or household head from each household to record biological relationships among all household members and to identify other first- or second-degree relatives living in the study villages, thereby identifying genetic links between households. Households within the same family compound shared the same location and peri-domiciliary environment and as such, were treated as single units. Individuals were defined as belonging to the same extended pedigree if they were related to anyone else in the pedigree or were married to anyone in the pedigree. Pedigrees were assembled and indexed using PEDSYS (Dyke) and visualized using Cranefoot (Southwest Foundation for Biomedial Research, San Antonio, TX).^32^ The number of informative relative pairs was determined using the relpairs routine of the software package SOLAR.^33^

High-resolution (0.6 m) QuickBird satellite data (dated October 16, 2003) were used for geo-referencing roads and potential mosquito-breeding sites. Geographic data were compiled, and maps were created using ArcGIS 9.2 (Environmental Systems Research Institute Inc., Redlands, CA).

### Analysis.

The phenotype under study was the density of blood stage *Plasmodium* infection. Parasite densities from all individuals, regardless of clinical malaria status and reported use of medication in the preceding weeks, were included in the analyses. Heritability estimation was undertaken using variance-component analysis to estimate the relative contribution of (1) additive genetic effects, (2) domestic environmental effects, (3) individual- and household-level covariates, and (4) unexplained residual variation to total variation (V_T_) in density of *Plasmodium* spp. parasitaemia. In brief, this approach incorporates information on genetic relationships within and between households to partition total phenotypic variation into its genetic, household, and other causes. Incorporation of a relationship matrix into a random-effect model, built from the pedigree described above and containing the expected degree of genetic relationship between all participants in the study, thus allows the covariance between relatives to depend on the degree of relatedness between them, providing an estimate of the total cumulative effect of individual genes (the additive genetic effect) on phenotypic variation. This is essentially a generalized animal model, an established quantitative genetic method that has been used extensively to model biological and disease phenotypes.^34^,^35^

Models were estimated by means of a maximum likelihood-based variance-decomposition approach implemented in the computer package SOLAR 4.2.0.^33^ Because this most frequent approach does not allow for non-normal distributions of the trait, the parasite-density data were logarithmically transformed before analysis (i.e., ln [density + 1]). Covariates tested included individual-level covariates (age, sex, reported medication history, and bed-net usage), household characteristics (household construction and crowding, income, education of household head and primary caregiver, relative socio-economic status, and bed-net ownership and type), and residential location (distance from geographical features). All covariates were included in an initial full model, and non-significant (*P* < 0.1) covariates were excluded sequentially to generate a minimal adequate model. Excluded covariates were retested in the minimal model to confirm lack of significance.

Subsequently, a series of nested multivariate mixed-effect linear-regression models were investigated. The sporadic model attributes variation in levels of *Plasmodium* parasitaemia entirely to a random, individual-specific effect (*e*^2^). The household model introduces an additional household-level random effect (*c*^2^), allowing estimation of the effect of unmeasured factors associated with the common domestic environment, whereas the polygenic model introduces a random effect describing additive genetic variation (*h*^2^; heritability). Finally, in the saturated model, all of these effects are modeled and estimated together. The variance parameters were standardized by dividing by the total phenotypic variation, V_T_ (i.e., the proportion of total variance attributable to each parameter was estimated).

To determine whether there were differences in heritability of density of *Plasmodium* parasitaemia between children (< 16 years) and adults (≥ 16 years), separate variance components were fit for each age group using the approach of Towne and others and Breitling and others.^36^,^37^ This approach can be considered a special case of a bivariate trait model in which no subject has observations for both traits (i.e., child and adult parasite counts). A series of models were fit in which variance components (*h*^2^ and *e*^2^) were constrained to be equal for both age groups or were allowed to vary between children and adults. χ^2^ testing based on likelihood ratios was then used to compare nested models and test whether variance components differed significantly between age groups. Because log-parasite density in adults showed residual kurtosis of more than 0.8, the *tdist* option, which creates an extra parameter in the model to describe the distribution of the phenotype, was applied in all analyses. The relative contribution of fixed covariates to total phenotypic variance in the final model was estimated by comparing the trait standard deviation (SD) in models with and without fixed covariates.

## Results

Of the 2,334 individuals for whom family structure was established, 1,711 provided household information and parasitological data. This represents 72.6% of the enumerated population in the four study villages. Comparison with the total enumerated population showed that the final study sample undersampled adult males (*P* < 0.001). There were, however, no statistical differences in the size or relative socio-economic status of participant and non-participant households.^30^ There were 341 compounds, with 1–19 (median = 4) phenotyped individuals per compound; an average compound comprised 2.9 phenotyped children (0–15 years). Table 1 describes the number of relative pairs included in these analyses. In total, when considering up to 8 degrees of relatedness, there were 12,440 informative relative pairs within the study population available for analysis.

### Parasitological and demographic data.

Overall, prevalence of *Plasmodium* infection was 38.5% (geometric mean density = 914 parasites/μL). Parasite density peaked between 3 and 5 years (geometric mean = 2,405 parasites/μL) and declined throughout childhood and adolescence. Total variance (V_T_) in log-density was 14.17 in children (< 16 years), falling to 5.21 in adults (≥ 16 years). There was no clear sex dependency. Findings from preliminary linear-regression models of logistically transformed density (i.e., ln (density + 1)) reflect the negative binomial regression analysis presented in the accompanying paper^30^; log-density was associated with bed-net use, household crowding, and proximity to rice paddies and the central rocky area. Of note, density of parasitaemia did not differ significantly for those who reported receiving anti-malarial medication in the preceding month (*N* = 1,046; 56.7% of the participant population).

### Household and genetic determinants (general and age-stratified analysis).

After adjusting for associated covariates when modeling parasite log-density, the effects of household and familial clustering could not be separated for this study population (models shown in Supplemental Appendix S1, available at www.ajtmh.org). To explore whether this may result from an age–genotype interaction (i.e., the role of genetic effects may not remain constant throughout life), analysis was repeated after age stratification (< 16 years and ≥ 16 years); data for 5,586 and 214 informative relative pairs were available for children and adults, respectively. Covariates included in the child model were age group, bed-net use, education level of the household head, and residential location (proximity to the central rocky area); the adult model was adjusted for age group and residential location (proximity to rice-growing area). In children, the data were best described by a polygenic model, with no household clustering of infection (χ^2^; *P* < 0.001); variation in density in adults was best described by a household model, with no additive genetic effect (χ^2^; *P* = 0.09) (Supplemental Appendix S2, available at www.amtmh.org).

Bivariate outcome models were, therefore, developed to test whether variance components differed significantly between age groups. Results from models fitting general and age-specific variance components are shown in Table 2, and details of the important likelihood ratio tests discussed below are shown in Table 3.

Estimates for both *h*^2^ and *c*^2^ showed pronounced age differences in models, allowing these parameters to vary freely between age groups, with estimates of additive genetic parameters being substantially higher in children than in adults. In the saturated model (model 7), heritability of parasite log-density in children was 0.293 compared with 0.024 in adults, whereas the proportion of variance explained by household effects was 0.005 in children and 0.039 in adults. Refitting the polygenic model with *h*^2^ constrained to be the same for both age groups (model 3; i.e., a general additive genetic effect) indicated a significantly improved model fit for age-specific polygenic models (*P* = 0.03); the significance of the age-specific genetic effect remained when controlling for either a general or age-specific household component (*P* = 0.04). In contrast, after controlling for an additive genetic effect (general or age-specific *h*^2^), there was no evidence of a significant difference between age-specific household effects (*c*^2^).

Thus, based on the sequence of results presented above, age-specific models incorporating additive genetic effects (for children only) and household-effect components (for adults only) emerged as the most realistic reflection of the underlying structure of the density of *Plasmodium* parasitaemia. Final estimates of all fixed effects and variance parameters are shown in Table 4. When stratifying by age group, only 3–4% of total variation in log-density could be explained by age and bed-net use combined. Household location (and income, for children) explained 1–2% of variation in *Plasmodium* log-density, with a further 5% of variation in adult densities explained by unmeasured domestic environmental factors. There was no evidence of household clustering of the level of malaria parasitaemia in children after taking into account the contribution of genetics, which was moderately high at 26%. Evidence for this genetic involvement, however, did not remain in adulthood. Overall, 70% of variation in malaria burden remained unexplained in children, and 91% remained unexplained in adults.

## Discussion

Analyses presented here focus on the influence of familial and household factors on *Plasmodium* infection levels in a rural Ugandan population after controlling for individual characteristics and environmental effects. Initial results suggested that heritability for asymptomatic *Plasmodium* parasitaemia (log-density) was as low as 5%. More detailed modeling allowing for age-dependent effects, however, revealed striking differences in heritability and other sources of between-individual variation in malaria parasitaemia between children (< 16 years) and adults (≥ 16 years). Results indicate that, in children, density of *Plasmodium* parasitaemia in this Ugandan community is controlled to some degree by additive genetic factors, which account for approximately one-quarter of the phenotypic variation. This is remarkably consistent with previous quantitative genetic studies conducted in several epidemiological settings.^8^,^9^,^28^,^29^ In contrast, there was no evidence for genetic control of parasitaemia in adults.

There is a remarkable similarity between heritability of *Plasmodium* parasite density reported here for children (26%) and previous observations for asymptomatic parasite density and/or parasite density during clinical episodes in population cohorts from Thailand, Sri Lanka, Tanzania, and Senegal.^8^,^9^,^28^,^29^ The relative similarity of these heritability estimates (which range between 10% in Tanzania and 33% in Senegal) is noteworthy given the very different transmission intensities, socio-demographic characteristics, and genetic backgrounds existing in these populations, and it clearly suggests a genetic contribution to the control of peripheral blood-parasitaemia levels, at least in childhood. Numerous population studies have confirmed the important protective role of inherited blood disorders that occur regionally,^38^ although genotype data are not available for quantification of their involvement here. However, an increasing number of studies conducted both in the field and in the laboratory^39^,^40^ suggest that, on their own, even the most prominent of the known malaria-resistance genes make only minor contributions to total genetic variation; instead, evidence suggests that malaria resistance is under complex multigenic control.^7^,^23^

The genetic effects for malaria parasitaemia observed here seem to fundamentally differ between children and adults (defined as ≥ 16 years), pointing to a declining influence of genetics on the control of parasite density with age in this population. To our knowledge, the bivariate variance-component approach adopted here (which has the inherent advantage of allowing statistical tests of the significance of observed differences in heritability) represents a new approach for investigating age–genotype interactions in the control of *Plasmodium* parasitaemia, although the approach has been used previously to investigate age and sex differences in heritability of human hookworm infection.^37^ Previously, segregation analyses have also suggested the existence of strong interactions between age and putative major genetic effects controlling blood-infection levels in populations living in Cameroon^23^ and Burkina Faso,^24^ and similar observations have likewise been made for infection with lymphatic filariasis in Indonesia using alternative statistical methods.^41^ It is important, however, to recognize that microscopy may have been insufficiently sensitive to detect significant differences in parasitaemia between adults, who primarily present low-grade infections when infected. As such, a higher discriminating tool such as real-time polymerase chain reaction (PCR) may have proved a more accurate measure of parasite density.^42^ There were also considerably fewer informative relative pairs available for the adult group, reducing the power to separate household and genetic effects. Nevertheless, these results clearly suggest that putative genetic differences are easier to detect in children than in adults.

Genetic differences between individuals may play an important biological role during the development of anti-malarial immunity in childhood. For example, genetic contributions to acquired immunity have been previously shown,^43^,^44^ with reported differences in the heritability of antibody isotypes and subclass responses in children and adults.^45^ Based on this hypothesis, we would expect heritability to be higher in children than adults. It is perhaps surprising, however, that genetic contributions exerted through innate immunity pathways and red blood cell polymorphisms^2^,^38^ were not apparent in the adult population in this study, although these genetic influences may also act primarily in children before immunity becomes important. Differences between children and adults may alternatively be explained by simple differences in exposure: if exposure is consistently high in children, heritability will seem higher in this group, whereas highly heterogeneous environmental exposures in adults may mask the effect of genetic factors. Lastly, it should be noted that the absence of observable heritability may simply be attributed to a lack of power.

One pertinent difference between this and other studies of heritability of malaria is the relative influence of the household. We observed only a marginal effect of house on *Plasmodium* densities in adults and no effect on densities in children. Substantial household clustering of *P. falciparum* cases has previously been observed in genetic studies in Sri Lanka^8^ and Kenya.^7^ However, clinical episode parasite-density phenotypes in Sri Lanka^8^ and Thailand^9^ were not influenced by household, and previous quantitative genetic studies of asymptomatic parasite densities have not included household effects. Taken together, these results suggest that whereas household and environmental effects may contribute to the tendency to become infected (i.e., they influence the risk of being bitten by an infective mosquito), they may impact less on the parasite after the infection has started (i.e., they have less influence on the host response to infection). These findings are, however, in disagreement with a study conducted in a low-transmission setting in Senegal, in which behavioral and environmental risk factors associated with the household were seen to significantly reduce clustering of blood-parasitaemia levels within nuclear families.^46^ There may, however, have been insufficient variation in exposure between households in our high-transmission study site to cause substantial environmental clustering of infection.

When interpreting the presented results, it is important to recognize that heritability is a population-specific measure, and our estimates are likely to be conservative. The feasibility of this approach to statistically separate the contributions of different variance components also depends on the sample size, pedigree and household structures, and available data for potentially confounding covariates, although studies do suggest that the impact of pedigree structure on precision and accuracy of heritability estimates may be minimal for purely additive genetic effects.^47^ Although care was taken to ensure accuracy, we acknowledge that assessment of paternity was subject to some reporting error, reducing our observed estimates of heritability.^48^ Using household effects as a surrogate measure of shared environmental factors is also unlikely to be completely accurate,^49^ and partitioning variance between these effects is difficult. Lastly, some genetic effects, particularly dominance effects, can be difficult to separate from shared household environment.^37^ Results also represent a single time point, but parasite densities are known to fluctuate from day to day^50^ and even with time or day,^51^ and as such, these findings can only provide a relatively limited view of a highly dynamic system. This represents a potential limitation; studies incorporating repeat measurements may yield different results. It is likely, however, that such fluctuation will be non-systematic and thus, will only contribute to decreased household and genetic variance components by increasing unexplained error. It is, therefore, expected that sampling on multiple occasions would increase observed heritability or at least, provide greater precision in variance-component estimates.

In conclusion, these results clearly show that there are major human genetic effects influencing asymptomatic *Plasmodium* infection in children in this population, suggesting that genetics plays a determinant role in the outcome of an infection before the development of immunity. In contrast, differences in domestic environmental exposure play a lesser role in this area of high (and stable) transmission. Large genome-wide association studies of children with differing parasite densities and different antibody-response profiles should allow identification of the genetic determinants of naturally acquired immunity,^52^ leading to a better understanding of the development of host resistance and ultimately, a better vaccine design and use.

## Supplementary Material

Appendix Tables

## Figures and Tables

**Table 1:** Distribution of informative relative pairs by degree of relationship

Relationship	Degree of relatedness	Number of informative pairs
Parent–offspring	1	1,476
Sibling	1	1,308
Half sibling	2	238
Grandparent–grandchild	2	839
Avuncular	2	1,021
Cousin	3	1,584
Half avuncular	3	444
Grand avuncular	3	346
Great grandparent	3	114
Higher-degree relative	4–7	5,070
Total		12,440

**Table 2:** Age-specific variance component analysis of heritability (*h*^2^) and household effects (*c*^2^) of *Plasmodium* log-density

Model	Ln*L*	*h*^2^			*c*^2^			*e*^2^		
		All	Children	Adults	All	Children	Adults	All	Children	Adults
1. Sporadic (*e*^2^)	−2,746.0	–	–	–	–	–	–	1	–	–
2. Polygenic (*h*^2^* + *e*^2^)	−2,737.2	0.220	–	–	–	–	–	0.078	–	–
3. Polygenic (*h*^2^*+ e*^2^)	−2,735.4	–	0.304	0.069	–	–	–	–	0.696	0.078
4. Paturated (*h*^2^* + c^2^* *+ e*^2^)	−2,736.8	0.169	–	–	0.290	–	–	0.802	–	–
5. Saturated (*h*^2^* *+ c*^2^*+ e*^2^)	−2,736.6	0.146	–	–	–	0.051	0.015	–	0.803	0.839
6. Saturated (*h*^2^*+ c*^2^* *+ e*^2^)	−2,735.2	–	0.266	0.044	0.019	–	–	–	0.715	0.938
7. Saturated (*h*^2^*+ c*^2^*+ e*^2^)	−2,735.0	–	0.293	0.024	–	0.005	0.039	–	0.702	0.936

Comparison of models in which *h*^2^ and/or *c*^2^ are constrained to be equal for both age groups (indicated by an asterisk) or allowed to vary between adults and children. For hypothesis testing, see Table 3. Children are aged < 16 years (5,586 relative pairs), and adults are ≥ 16 years (214 relative pairs).

**Table 3:** Likelihood ratio tests for selected models presented in Table 2

Models	Alternative hypothesis	DF	*P*
2 vs. 3	*h*^2^ differs between age groups (not controlling for household)	1	0.03
4 vs. 6	*h*^2^ differs between age groups (when *c*^2^_child_ = *c*^2^_adult_)	1	0.04
4 vs. 5	*c*^2^ differs between age groups (when *h*^2^_child_ = *h*^2^_adult_)	1	0.3
5 vs. 7	*h*^2^ differs between age groups (when *c*^2^_child_ ¹ *c*^2^_adult_)	1	0.04
6 vs. 7	*c*^2^ differs between age groups (when *h*^2^_child_ ¹ *h*^2^_adult_)	1	0.3

Comparison of models in which *h*^2^ and/or *c*^2^ are equal for both age groups or allowed to differ between adults and children.

DF = degrees of freedom.

**Table 4:** Final estimates for covariates and variance components for malaria infection in Mulanda

Explanatory variables	*Plasmodium* ln (density + 1)			
	Children	Children SE	Adults	Adult SE
Intercept	2.95	0.34	2.10	0.23
Individual characteristics				
Age group (years)				
< 2	1	–		
3–4	1.27	0.37		
5–9	1.45	0.33		
10–15	0.57	0.35		
16–24			1	–
25–49			−0.76	0.20
≥ 50			−1.16	0.22
Slept under a net last night	−0.65	0.27		
Household characteristics				
Educated household head*	1.76	0.57		
> 500 m from rocky area	0.48	0.28		
> 750 m from paddy fields			−0.52	0.21
Variance components as proportions of residual variation				
Additive genetic	0.26	0.08	0†	–
Shared domestic environment	0†	–	0.05	0.04
Individual-specific	0.74	0.08	0.95	0.04

Covariate and variance estimates for *Plasmodium* parasitaemia (stratified by age group: children < 16 years; adults ≥ 16 years), including standardized variance parameter estimates.

* Primary career with any level of education (primary, incomplete, and above).

† Variance parameter restricted to zero.
