# Supplementary material for: Heritability of Plasmodium Parasite Density in a Rural Ugandan Community
Source: Am J Trop Med Hyg. 2010 Nov 5;83(5):990–5. doi: 10.4269/ajtmh.2010.10-0049 (PMC2963957; doi:10.4269/ajtmh.2010.10-0049)
Supplement: Appendix Tables [file SD1.pdf]

Appendix S1

Variance component analysis of heritability (h^2) and household effects (c^2) of density of asymptomatic *Plasmodium* parasitaemia showing standardized variance parameter estimates and estimated log-likelihood values for models with and without covariates (χ^2 and *P* value for comparison of models)

	Variance component estimates					
Model	Ln*L*	h^2	c^2	e^2	χ^2 test	*P*
No covariates						
1. Sporadic (e^2)	−3,002					
2. Polygenic ($h^2 + e^2$)	−2,998	0.080		0.920	A (2 vs. 1)	0.002
3. Household ($c^2 + e^2$)	−2,999		0.046	0.954	B (3 vs. 1)	0.003
4. Saturated ($h^2 + c^2 + e^2$)	−2,997	0.000	0.046	0.954	C (4 vs. 3)	0.5
					D (4 vs. 2)	0.04
With covariates*						
1. Sporadic (e^2)	−2,803			1		
2. Polygenic ($h^2 + e^2$)	−2,796	0.133		0.867	A (2 vs. 1)	< 0.001
3. Household ($c^2 + e^2$)	−2,796		0.062	0.938	B (3 vs. 1)	< 0.001
4. Saturated ($h^2 + c^2 + e^2$)	−2,795	0.053	0.041	0.906	C (4 vs. 3)	0.2
					D (4 vs. 2)	0.1

χ^2 test of nested models. A: $h^2 \neq 0$ (not controlling for c^2); B: $c^2 \neq 0$ (not controlling for h^2); C: $h^2 \neq 0$ (after controlling for c^2); D: $c^2 \neq 0$ (after controlling for h^2).
*Adjusted for age (categorical covariate), sex, bed-net use, education level, and residential location.

Appendix S2

Age-specific variance component analysis of heritability (h^2) and household effects (c^2) of asymptomatic *Plasmodium* parasitaemia testing for familial and household clustering

	Variance component estimates											
	Children (< 16 years)						Adults (≥ 16 years)					
Models	Ln*L*	h^2	c^2	e^2	χ^2 test	*P*	Ln*L*	h^2	c^2	e^2	χ^2 test	*P*
No covariates												
1. Sporadic (e^2)	−1,742.9						−1,003.1					
2. Polygenic ($h^2 + e^2$)	−1,733.1	0.304		0.606	A (2 vs. 1)	< 0.001	−1,002.8	0.076		0.024	A (2 vs. 1)	0.2
3. Household ($c^2 + e^2$)	−1,735.5		0.112	0.888	B (3 vs. 1)	< 0.001	−1,002.2		0.052	0.948	B (3 vs. 1)	0.09
4. Saturated ($h^2 + c^2 + e^2$)	−1,733.1	0.304	0.006	0.880	C (4 vs. 3)	< 0.001	−1,002.1	0.025	0.048	0.927	C (4 vs. 3)	0.4
					D (4 vs. 2)	0.5					D (4 vs. 2)	0.12
With covariates†												
1. Sporadic (e^2)	−1,718.2						−986.4			1		
2. Polygenic ($h^2 + e^2$)	−1,710.7	0.261		0.739	A (2 vs. 1)	< 0.001	−986.1	0.081		0.919	A (2 vs. 1)	0.2
3. Household ($c^2 + e^2$)	−1,712.7		0.096		B (3 vs. 1)	< 0.001	−985.5		0.050	0.095	B (3 vs. 1)	0.09
4. Saturated ($h^2 + c^2 + e^2$)	−1,710.7	0.261	0.004	0.735	C (4 vs. 3)	< 0.001	−985.4	0.028	0.046	0.926	C (4 vs. 3)	0.4
					D (4 vs. 2)	0.5					D (4 vs. 2)	0.12

Children are aged < 16 years (5,586 relative pairs), and adults are aged ≥ 16 years (214 relative pairs). For children, the best model was the saturated model (familial and household clustering); for adults, the best models were sporadic and household (i.e., weak evidence of household clustering). χ^2 test of nested models. A: $h^2 \neq 0$ (not controlling for c^2); B: $c^2 \neq 0$ (not controlling for h^2); C: $h^2 \neq 0$ (after controlling for c^2); D: $c^2 \neq 0$ (after controlling for h^2).
† Child model was adjusted for age group (< 2, 3–4, 5–9, and 10–15 years), bed-net use, education of household head, and proximity to rocky areas; adult model was adjusted for age (15–25, 25–49, and ≥ 50 years) and proximity to rice-growing area.
